# BAL Flow Cytometry Unmasks Nodal T Follicular Helper Cell Lymphoma–Associated Hemophagocytic Lymphohistiocytosis When Bone Marrow Is Nondiagnostic: A Case Report

**DOI:** 10.1155/crpu/7093340

**Published:** 2026-05-27

**Authors:** Mwelwa Chizinga Mwelwa

**Affiliations:** ^1^ Department of Pulmonary Medicine, Orlando Health Medical Center, Orlando, Florida, USA

**Keywords:** bronchoalveolar lavage, case report, flow cytometry, hemophagocytic lymphohistiocytosis, T follicular helper cell lymphoma

## Abstract

Hemophagocytic lymphohistiocytosis (HLH) is a life‐threatening hyperinflammatory syndrome that often presents with nonspecific pulmonary manifestations, frequently mimicking severe pneumonia or acute respiratory distress syndrome (ARDS). We report a case of malignancy‐associated HLH secondary to nodal T follicular helper (TFH) cell lymphoma presenting as nonresolving pneumonia with progressive hypoxemic respiratory failure. Bronchoalveolar lavage (BAL) flow cytometry demonstrated an aberrant CD4‐predominant T‐cell population (CD4:CD8 ratio 9.2:1) with loss of pan‐T cell antigens CD5 and CD7, providing early evidence of pulmonary lymphoma involvement and prompting expedited hematological evaluation. Critically, bone marrow flow cytometry was nondiagnostic despite histological involvement, highlighting BAL as the higher yield diagnostic site. This case argues for routine BAL immunophenotyping in critically ill patients with nonresolving pulmonary infiltrates, hyperferritinemia, and cytopenias.

## 1. Introduction

Hemophagocytic lymphohistiocytosis (HLH) is a hyperinflammatory syndrome characterized by excessive immune activation, cytokine storm, and multi‐organ dysfunction. This hyperinflammatory state results from failure of normal regulatory suppression of macrophages and lymphocytes, producing unchecked inflammation that leads to organ damage [1]. HLH can be primary (genetic) or secondary; secondary HLH in adults is frequently triggered by infection, autoimmune disease, or malignancy [2]. T‐cell lymphomas, particularly those of T follicular helper (TFH) cell origin—reclassified as a distinct entity in the 2022 WHO Classification of Hematolymphoid Tumors [3]—are uniquely prone to triggering HLH due to their inherent capacity for cytokine secretion and immune dysregulation [4].

Pulmonary involvement is common in HLH, reported in up to 42% of adult cases [5]. Pulmonary symptoms may dominate the clinical presentation, leading to diagnostic anchoring bias toward infection and delayed recognition of HLH. Early identification is critical, as malignancy‐associated HLH carries high mortality without prompt treatment. We describe a pulmonary‐predominant presentation of nodal TFH cell lymphoma–associated HLH in which bronchoalveolar lavage (BAL) flow cytometry provided the critical early diagnostic clue to the underlying malignancy, notably preceding a nondiagnostic bone marrow flow cytometry result.

## 2. Case Presentation

A 46‐year‐old male presented with progressive dyspnea, fatigue, high‐grade fevers, and nonproductive cough. He had no chronic pulmonary history prior to the current illness. His clinical course had been subacute over approximately 2 months from symptom onset to the index ICU admission, characterized by recurrent cough, shortness of breath, and fevers requiring multiple hospitalizations for presumed pneumonia. Despite transient improvement with empiric antibiotics and corticosteroids, he developed worsening hypoxemia requiring admission to the intensive care unit (ICU). Bronchoscopy with BAL was performed during this ICU admission, approximately 2 months after initial symptom onset.

On admission, vital signs were notable for a temperature of 39.0°C, heart rate of 115 beats/min, respiratory rate of 32 breaths/min, and blood pressure of 116/80 mmHg. Oxygen saturation was 95% on high‐flow nasal cannula (HFNC; 40 L/min, FiO^2^ 60%). Physical examination revealed diffuse bibasilar crackles; there was no palpable lymphadenopathy.

### 2.1. Laboratory Findings

Initial laboratory evaluation revealed leukopenia (WBC 2.4 × 10^3^/*μ*L, with an absolute neutrophil count of 0.5 × 10^3^/*μ*L), anemia (hemoglobin 10.7 g/dL), and normal platelets (152 × 10^3^/*μ*L). Liver enzymes were markedly elevated (AST 624U/L, ALT 471 U/L, and alkaline phosphatase 244 U/L) with hypoalbuminemia (2.0 g/dL). Ferritin was profoundly elevated at 17,501 ng/mL. Triglycerides were elevated (256 mg/dL), and fibrinogen was low at 184 mg/dL. By hospital Day 3, hemoglobin had declined to 8.7 g/dL, confirming progressive cytopenias. The calculated HScore was 223 out of 337, corresponding to a 96% probability of HLH [6]. Clinical and laboratory findings at presentation are summarized in Table 1.

**Table 1:** Clinical and laboratory findings at presentation. HScore calculated per Fardet et al. [6] at admission, prior to bone marrow biopsy results (hemophagocytosis scored as 0 at the time of initial calculation).

Parameter	Value	Reference range
Demographics		
Age/sex	46 years/male	*—*
BMI	18.3 kg/m^2^	*18.5–24.9*
Hematology		
White blood cells	2.4 × 10^3^/*μ*L	*4.5–11.0*
Hemoglobin	10.7 g/dL	*13.5–17.5*
Platelets	152 × 10^3^/*μ*L	*150–400*
Hepatic panel		
AST	624 U/L	*10–40*
ALT	471 U/L	*7–56*
Alkaline phosphatase	244 U/L	*44–147*
Albumin	2.0 g/dL	*3.5–5.5*
HLH‐specific markers		
Ferritin	17,501 ng/mL	*20–250*
Triglycerides	256 mg/dL	*< 150*
Fibrinogen	184 mg/dL	*200–400*
HScore (calculated)	223/337	*≥ 168 = HLH likely*
HScore probability of HLH	96%	*—*

### 2.2. Imaging

**Figure 1:**
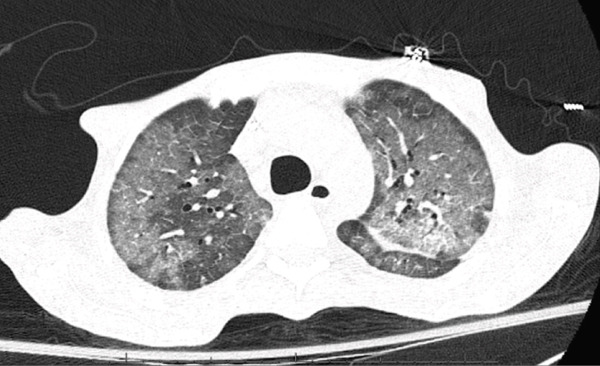
CT chest demonstrating diffuse bilateral ground‐glass opacities with confluent consolidations in the lower lobes and extensive mediastinal lymphadenopathy.

**Figure 2:**
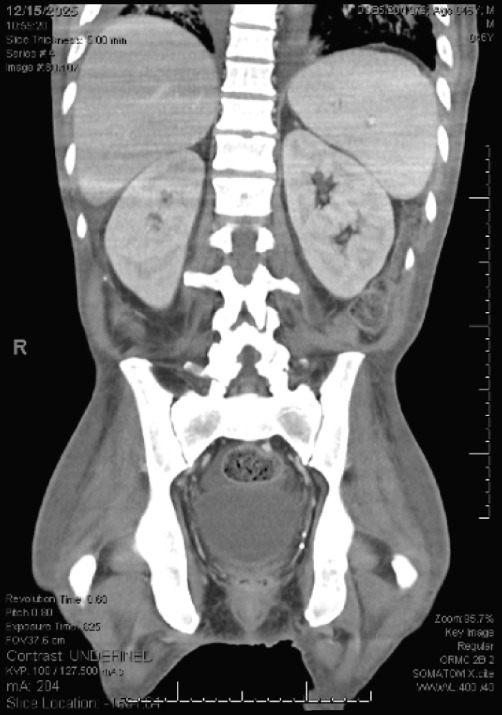
CT abdomen and pelvis (coronal view) demonstrating splenomegaly (138 mm).

Computed tomography (CT) of the chest (Figure 1) demonstrated diffuse bilateral patchy ground‐glass opacities with confluent consolidations, particularly in the lower lobes, and extensive mediastinal and axillary lymphadenopathy. CT of the abdomen and pelvis revealed diffuse mesenteric, retroperitoneal, iliac, and inguinal lymphadenopathy with splenomegaly measuring 13.8 cm (Figure 2).

### 2.3. Bronchoscopy and BAL Findings

Bronchoscopy with BAL was performed to evaluate for opportunistic infection and noninfectious etiologies of nonresolving pulmonary infiltrates. BAL differential cell count demonstrated a lymphocyte‐predominant pattern (62% lymphocytes, 28% macrophages, 8% neutrophils, and 2% eosinophils). Flow cytometric immunophenotyping of the BAL lymphoid population revealed an aberrant T‐cell population; findings are summarized in Table 2.

**Table 2:** Bronchoalveolar lavage (BAL) flow cytometry immunophenotyping results. CD4:CD8 ratio markedly elevated beyond the normal reference range (1–3.5:1) and beyond the sarcoidosis‐associated range (typically 3.5–5:1). Loss of CD5 and CD7 on CD4+ T cells is characteristic of T‐cell neoplasia. PD‐1 and dim CD10 expression support TFH cell origin.

Parameter	Value	Interpretation
BAL differential		
Total lymphocytes	62%	*Markedly elevated (normal < 15%)*
Macrophages	28%	*Relatively reduced*
Neutrophils	8%	*Within normal range*
Eosinophils	2%	*Within normal range*
T‐cell immunophenotype		
CD3+ (pan‐T cell)	78% of lymphocytes	*Confirms T-cell predominance*
CD4+	71% of lymphocytes	*Markedly elevated*
CD8+	7.7% of lymphocytes	*Reduced*
CD4:CD8 ratio	9.2:1	*Markedly elevated (normal 1–3.5)*
Aberrant antigen expression		
CD5 (pan‐T cell)	Loss on 68% of CD4+ cells	*Aberrant—suggests T-cell neoplasm*
CD7 (pan‐T cell)	Loss on 74% of CD4+ cells	*Aberrant—suggests T-cell neoplasm*
CD10	Dim on 22% of CD4+ cells	*Consistent with TFH origin*
PD‐1 (CD279)	Positive on 45% of CD4+ cells	*Supports TFH phenotype*

The BAL findings were interpreted as highly suggestive of a T‐cell lymphoproliferative disorder with TFH immunophenotype. The markedly elevated CD4:CD8 ratio of 9.2:1 far exceeded the normal BAL reference range (1–3.5:1) and even surpassed the range typically associated with sarcoidosis (3.5–5:1) and, combined with CD5/CD7 loss on the majority of CD4+ cells, provided the first objective evidence of malignancy in this case. Microbiological cultures from BAL were negative for bacterial, fungal, and mycobacterial organisms. *Pneumocystis jirovecii* PCR was negative.

### 2.4. Bone Marrow and Lymph Node Evaluation

Bone marrow biopsy revealed a hypercellular marrow (70% cellularity) with approximately 20% atypical T cells consistent with a mature T‐cell lymphoproliferative disorder on histological examination. Rare hemophagocytic histiocytes were identified, supporting the diagnosis of HLH. Notably, bone marrow flow cytometry did not demonstrate an overt clonal T‐cell population, likely reflecting patchy marrow involvement with insufficient neoplastic cells in the aspirate to exceed the sensitivity threshold of flow cytometry (typically 0.1%–1% of total events).

Excisional biopsy of a right inguinal lymph node confirmed a mature CD4‐positive T‐cell lymphoma. Immunohistochemistry (IHC) demonstrated CD3, CD4, CD5, and CD7 positivity, with PD‐1 and BCL6 expression in a subset of T cells and ICOS positivity. The overall architectural and immunophenotypic pattern was diagnostic of nodal TFH cell lymphoma, NOS, per the 2022 WHO Classification [3].

### 2.5. Treatment and Clinical Course

Given the HScore of 223 (96% probability of HLH), treatment was initiated with HyperCY (cyclophosphamide/dexamethasone) as combined HLH‐directed and lymphoma‐directed therapy. However, this regimen was curtailed by worsening acute liver failure, attributed to both HLH‐mediated hepatic inflammation and direct lymphomatous infiltration.

Therapy was subsequently escalated to dose‐reduced CHOEP (cyclophosphamide, doxorubicin, vincristine, etoposide, and prednisone), a first‐line regimen for nodal TFH lymphomas [7]. Given identification of CCR4 expression on the neoplastic T cells, mogamulizumab—an anti‐CCR4 monoclonal antibody with demonstrated activity in CCR4‐positive peripheral T‐cell lymphomas [8]—was combined with CHOEP. Both regimens yielded only transient improvement in fevers and liver enzymes before disease recrudescence.

Following transfer to a tertiary cancer center, salvage therapy was planned with duvelisib (a PI3K‐*δ*/*γ* inhibitor) combined with azacitidine targeting epigenetic dysregulation—a hallmark of TFH lymphomas, which harbor frequent *TET2* and *DNMT3A* mutations [9]. Due to insurance barriers precluding duvelisib access, romidepsin—a histone deacetylase (HDAC) inhibitor approved for relapsed peripheral T‐cell lymphoma—was substituted alongside etoposide for ongoing HLH control.

While the patient achieved a biochemical response with improvement in HLH parameters (declining ferritin and transaminases) and neutrophil recovery, he developed persistent refractory thrombocytopenia despite IVIG. Ultimately, he suffered progressively worsening respiratory failure and cytopenias and succumbed to disease complications.

## 3. Discussion

This case illustrates a pulmonary masquerade presentation of HLH in which progressive hypoxemic respiratory failure and diffuse pulmonary infiltrates initially suggested severe pneumonia. Pulmonary involvement is common in HLH and may dominate the initial presentation, leading to premature diagnostic closure on infection. A key factor that further delayed diagnosis was the partial, transient response the patient exhibited after receiving empiric corticosteroids and antibiotics for presumed pneumonia. Corticosteroids likely dampened the cytokine storm temporarily, creating a false impression of resolving infection. In reality, the diffuse consolidations reflected cytokine‐mediated alveolar injury and lymphocytic infiltration rather than primary infection.

The patient′s febrile illness, cytopenias, transaminitis, and eventual nonresponsiveness to empiric treatment prompted calculation of an HScore. The HScore is a weighted scale of nine clinical and laboratory variables that achieves 100% sensitivity and 91.4% specificity for HLH at a cutoff of 168 (range 0–337) [6]. Our patient′s HScore was 223, equating to a 96% probability of HLH and justifying immediate hematological intervention. Notably, soluble CD25 (sIL‐2R) and NK cell activity—two of the nine HScore variables—were not measured during the index hospitalization. This represents a limitation, as sCD25 elevation is among the most specific biomarkers for HLH, and its absence prevents full scoring of both the HScore and the HLH‐2004 criteria.

Regarding the HLH‐2004 diagnostic criteria [10], the patient satisfied the following: fever ≥ 38.5°C (temperature 39.0°C), splenomegaly (13.8 cm), cytopenias affecting two or more lineages (neutropenia, ANC 0.5 × 10^3^/*μ*L; anemia, hemoglobin nadir 8.7 g/dL on hospital Day 3, meeting the < 9 g/dL threshold), hemophagocytosis on bone marrow biopsy, and hyperferritinemia ≥ 500 ng/mL (ferritin 17,501 ng/mL)—five of the eight criteria, meeting the diagnostic threshold. Hypertriglyceridemia (256 mg/dL) and hypofibrinogenemia (184 mg/dL) approached but did not meet the formal cutoffs of ≥ 265 mg/dL and ≤ 150 mg/dL, respectively. Soluble CD25 and NK cell activity were not assessed. Thus, the patient definitively met five of eight HLH‐2004 criteria, satisfying the diagnostic standard, with one additional criterion borderline and two not evaluated.

The etiologies of HLH in adults vary by geographic location and study population. In a series of 162 adults with HLH, hematological malignancies (especially non‐Hodgkin lymphoma) were the most common trigger, accounting for 57% of cases [11]. An additional 25% were triggered by infections, whereas 4% had both malignancy and concomitant infection. This diagnostic overlap underscores the critical role of bronchoscopy in patients with pulmonary‐predominant symptoms, as it can simultaneously evaluate for opportunistic infection and malignancy.

### 3.1. BAL Flow Cytometry: The Key Diagnostic Contribution

The central teaching point of this case is the diagnostic utility of BAL flow cytometry in the specific clinical scenario of nonresolving pulmonary infiltrates with HLH physiology. While BAL immunophenotyping is routinely performed for suspected leukemia or sarcoidosis, it is underutilized in the evaluation of T‐cell lymphomas with pulmonary involvement. In this case, BAL flow cytometry revealed an aberrant CD4‐predominant T‐cell population (CD4:CD8 ratio 9.2:1) with loss of CD5 on 68% and CD7 on 74% of CD4+ cells (Table 2). This provided the first objective evidence of malignancy, preceding the nodal diagnosis and—critically—outperforming the bone marrow flow cytometry, which was nondiagnostic despite histological involvement on biopsy.

The superiority of BAL over marrow flow cytometry in this case likely reflects two factors. First, the lung was the site of active inflammation and lymphocytic infiltration, concentrating the neoplastic clone in the alveolar compartment and yielding a high proportion of aberrant T cells in the lavage specimen. Second, bone marrow involvement was patchy, with neoplastic cells intermixed with normal hematopoietic elements in a ratio insufficient for detection by standard flow cytometric gating. This discordance has important implications: in pulmonary‐predominant HLH, the lung may be the highest yield diagnostic site, and reliance solely on bone marrow assessment risks missing the malignant population.

### 3.2. Immunophenotypic Discordance Between BAL and Lymph Node

A notable observation is the apparent discordance between BAL flow cytometry, which demonstrated loss of CD5 and CD7 on the aberrant T‐cell population, and lymph node IHC, which demonstrated CD5 and CD7 positivity alongside CD3, CD4, PD‐1, BCL6, and ICOS. Several factors may account for this finding.

First, flow cytometry and IHC differ fundamentally in their sensitivity thresholds and interpretation. Flow cytometry quantifies surface antigen density on individual cells and can detect partial antigen loss (dim expression or loss on a subpopulation), whereas IHC is scored as positive or negative based on overall staining pattern—a population with 30%–40% of cells retaining CD5/CD7 expression may be scored as “positive” on IHC but show clear antigenic loss on flow cytometry. In our case, CD5 loss was demonstrated on 68% of CD4+ cells, meaning 32% retained expression—a proportion likely sufficient for an IHC “positive” interpretation.

Second, intratumoral immunophenotypic heterogeneity is well described in TFH lymphomas. The neoplastic TFH cells may exhibit variable antigen expression across different anatomic compartments, with the pulmonary microenvironment potentially selecting for or enriching subclones with more pronounced antigen downregulation. This phenomenon has been reported in other T‐cell lymphomas where extranodal sites demonstrate more aberrant phenotypes than the primary nodal disease [12].

Third, the 32% of CD4+ cells retaining CD5 expression in the BAL population may not represent antigen‐positive neoplastic cells but rather admixed reactive T cells. In the inflammatory milieu of HLH‐associated alveolitis, recruitment of non‐neoplastic CD4+ T cells into the alveolar space is expected. These bystander reactive T cells would retain normal pan‐T cell antigen expression, potentially accounting for the CD5‐positive fraction without invoking true immunophenotypic heterogeneity within the neoplastic clone itself.

This discordance reinforces rather than undermines the diagnostic value of BAL flow cytometry: it detected an aberrant population that conventional IHC interpretation might have partially obscured, and it did so before either the marrow or nodal biopsy results were available.

### 3.3. TFH Biology and Susceptibility to HLH

The specific diagnosis of nodal TFH cell lymphoma is clinically relevant to the HLH presentation. TFH lymphomas—formerly classified under the umbrella of angioimmunoblastic T‐cell lymphoma (AITL) and reclassified in the 2022 WHO Classification as a distinct category [3]—are derived from follicular helper T cells, which are physiologically distinct in their capacity to secrete IL‐21, IL‐4, and CXCL13 and to support B‐cell activation and germinal center formation. This inherent cytokine‐secretory phenotype makes TFH lymphomas particularly prone to triggering the cytokine storm and macrophage activation seen in HLH. In a recent French series, HLH was identified as a complication in 14%–18% of TFH lymphoma cases, substantially exceeding the rate in other peripheral T‐cell lymphoma subtypes [9]. The prognosis of TFH lymphoma–associated HLH remains poor, with 5‐year overall survival below 30% in published cohorts [9].

## 4. Conclusion

Pulmonary‐predominant HLH secondary to nodal TFH cell lymphoma can masquerade as nonresolving pneumonia. This case demonstrates that BAL flow cytometry can detect occult pulmonary lymphoma involvement even when bone marrow flow cytometry is nondiagnostic—a finding with direct implications for the diagnostic evaluation of critically ill patients with unexplained pulmonary infiltrates and hyperferritinemia. We propose that in high‐risk pulmonary presentations with systemic inflammation, BAL analysis should routinely include flow cytometry with a comprehensive T‐cell panel (CD3, CD4, CD5, CD7, CD8, CD10, and PD‐1) to accelerate the diagnosis of malignancy‐associated HLH. Notably, ICOS and BCL6—markers that confirmed TFH origin at lymph node biopsy in this case—have limited applicability in BAL flow cytometry given their predominantly intracellular or nuclear localization, and are therefore not included in the proposed panel.

## Funding

No funding was received for this manuscript.

## Ethics Statement

Informed consent was obtained from the patient′s next of kin for the publication of this case report and accompanying images. This report does not contain any personally identifying information.

## Consent

No written consent has been obtained from the patients as there is no patient.

## Conflicts of Interest

The authors declare no conflicts of interest.

## Data Availability

All data underlying the results are available as part of the article, and no additional source data are required.
